# Ovarian Cancer-Associated Ascites Have High Proportions of Cytokine-Responsive CD56bright NK Cells

**DOI:** 10.3390/cells10071702

**Published:** 2021-07-06

**Authors:** Cláudia Rodrigues Tonetti, Caroline Natânia de Souza-Araújo, Adriana Yoshida, Rodrigo Fernandes da Silva, Paulo César Martins Alves, Taís Nitsch Mazzola, Sophie Derchain, Luís Gustavo Romani Fernandes, Fernando Guimarães

**Affiliations:** 1 School of Medicine Sciences, University of Campinas, Rua Tessália Vieira de Camargo-126, Campinas CEP 13083-887, SP, Brazil; claudinha.tonetti@gmail.com (C.R.T.); caroline.natania@yahoo.com.br (C.N.d.S.-A.); rodrigoiverson@hotmail.com (R.F.d.S.); derchain@fcm.unicamp.br (S.D.); lgrf@fcm.unicamp.br (L.G.R.F.); 2 Centro de Atenção Integral à Saúde da Mulher (CAISM), Women’s Hospital José Aristodemo Pinotti, University of Campinas, Rua Alexander Fleming-101, Campinas CEP 13083-881, SP, Brazil; adriana122013@gmail.com; 3 Center for Investigation in Pediatrics, University of Campinas, Rua Tessália Vieira de Camargo-126, Campinas CEP 13083-887, SP, Brazil; cesar.imperio@gmail.com (P.C.M.A.); mazzolat@unicamp.br (T.N.M.)

**Keywords:** cytotoxic lymphocytes, STAT5, pS6, inhibitory checkpoint receptor, tumor microenvironment, ectonucleotidases

## Abstract

Ovarian cancer is the most lethal gynecological malignancy, with serous histotype as the most prevalent epithelial ovarian cancer (EOC). Peritoneal ascites is a frequent comorbidity in advanced EOC. EOC-associated ascites provide a reliable sampling source for studying lymphocytes directly from tumor environment. Herein, we carried out flow cytometry-based analysis to readdress issues on NK and T lymphocyte subsets in women with advanced EOC, additionally evaluating phenotypic modulation of their intracellular pathways involved in interleukin (IL)-2 and IL-15 signaling. Results depicted ascites as an inflammatory and immunosuppressive environment, presenting significantly (*p* < 0.0001) higher amounts of IL-6 and IL-10 than in the patients’ blood, as well as significantly (*p* < 0.05) increased expression of checkpoint inhibitory receptors (programmed death protein-1, PD-1) and ectonucleotidase (CD39) on T lymphocytes. However, NK lymphocytes from EOC-associated ascites showed higher (*p* < 0.05) pS6 phosphorylation compared with NK from blood. Additionally, in vitro treatment of lymphocytes with IL-2 or IL-15 elicited significantly (*p* < 0.001) phosphorylation of the STAT5 protein in NK, CD3 and CD8 lymphocytes, both from blood and ascites. EOC-associated ascites had a significantly (*p* < 0.0001) higher proportion of NK CD56bright lymphocytes than blood, which, in addition, were more responsive (*p* < 0.05) to stimulation by IL-2 than CD56dim NK. EOC-associated ascites allow studies on lymphocyte phenotype modulation in the tumor environment, where inflammatory profile contrasts with the presence of immunosuppressive elements and development of cellular self-regulating mechanisms.

## 1. Introduction

Ovarian cancer ranks eighth among cancer-related deaths among women; however, it is the most lethal gynecological malignancy. The lethality is a consequence of its initial asymptomatic development and the lack of specific early detection biomarkers, which often delays the diagnosis of cancer. Seventy-nine percent of women with ovarian cancer are diagnosed at advanced stages, resulting in a poor five years survival rate of 20–25%, the worst among all gynecological malignancies [1,2,3,4]. Ovarian cancer comprises multiple subtypes, 90% of them being of epithelial origin. Epithelial ovarian cancers (EOC) are additionally categorized based on their morphology and genetic characteristics [5,6]. The serous histotype comprises 70% of all EOCs, which is further classified in low-grade (LGS) and high-grade (HGS) serous. The HGS is the most aggressive subtype and accounts for two-thirds of all ovarian cancer deaths [7,8]. Although the LGS is less frequent than HGS, women with advanced or recurrent LGS have similar survival rates as women with HGS [9,10,11].

Ascites is the pathological accumulation of fluid in the peritoneal cavity. Although present in different pathological conditions, peritoneal ascites is a distinguishable feature of women with advanced EOC, which is frequently observed at first diagnosis, as well as in almost all cases of relapse [12,13,14,15]. Malignant cells may or may not be detectable in EOC-associated ascites, which may display different signaling molecules and cell types, such as fibroblasts, adipocytes, mesothelial, endothelial and inflammatory cells [15]. The presence of different lymphocyte subsets has also been reported in EOC-associated ascites, which, in addition, undergo phenotypic modulation in response to this peculiar cancer-related environment [16,17,18,19]. However, biological processes underlying presence, functional modulation and role of lymphocytes in ascites are still poorly understood.

Among lymphocytes present in EOC-associated ascites, the natural killer (NK) cells have been systematically addressed by our research group. NK cells are categorized among the group 1 innate lymphoid cells (ILC-group 1), which are comprised of lymphocytes provided of cytotoxicity and producers of interferon-gamma (IFN-γ) [20,21]. Conventional NK lymphocytes are typically involved in the immune defense against malignant or virally infected cells, acting directly against such altered cells, as well as contributing to the development of the adaptive response through the release of cytokines and interactions with other cells of the immune system [21,22]. NK cells are known by their ability to kill malignant cells and, as demonstrated in murine models of melanoma, prostate, breast and lung cancer, they do play a role in restraining metastatic spread of cancer [23,24,25,26]. Additionally, NK cells with high spontaneous cytotoxicity have been correlated with a reduced incidence of cancer in humans [27,28]. Thus, NK cells have widely been considered for treatment of cancer, including in the emerging therapies based on monoclonal antibodies (MAb), since NK cells can recognize antibody-opsonized target cells through CD16 (FcγRIIIa) molecules and eliminate them [29].

In humans, NK cells comprise up to 15% of the peripheral blood lymphocytes, where they can be differentiated from other lymphocyte populations by their cell surface molecular phenotype CD3-CD56+. Furthermore, relative expression of CD56 molecule allows to categorize NK cells in two subsets known as CD56^bright^ and CD56^dim^, in accordance with their relative emission of fluorescence intensity, when labeled with fluorochrome-conjugated MAb [22,30]. Although related in their origin, these two NK subsets have biological differences that helped to consolidate the knowledge that CD56^dim^ NK subset mediates cytotoxic antitumor responses, while CD56^bright^ plays a role in modulation of immune response. Indeed, CD56^bright^ NK cells are primarily cytokine producers (IFN-γ, tumor necrosis factor or TNF, granulocyte-macrophage colony stimulator factor or GM-CSF) and display lower antitumor cytotoxicity at rest when compared to the CD56^dim^ NK cells [22,30,31,32]. Nevertheless, these concepts have been challenged and recent studies showed that antitumor response of CD56^bright^ NK cells can be markedly enhanced by IL-15 [33,34]. All biological aspects of NK cells are regulated by cytokines that bind to receptor complexes on the cell membrane and activate intracellular signaling pathways based on phosphorylation of signal transducing and transcription-activating protein kinases. Among other cytokines, IL-2 and IL-15 have partially overlapping properties and are implicated in the development, survival and cytotoxicity of NK cells [35,36,37]. In NK cells, the JAK-STAT5 and mTOR-S6 pathways have been identified as central in the convergence of signals from the activating cytokines IL-2 and IL-15 and, therefore, involved in the functional modulation of these cells [38,39,40].

Studies addressing the phenotypic modulation of lymphocytes present in EOC-associated ascites have linked the impairment of antitumor response of NK cells with decreasing in the expression of DNAM-1-, NKp30-, CD16- and NKG2D-activating receptors. This effect was accounted to the downregulation of these activating receptors as a consequence of excessive exposure to their ligands in tumor environment [19,41,42]. Furthermore, the CA125 (cancer antigen-125 or MUC16) molecule, which is usually found elevated in plasma and ascites of women with advanced EOC, has been shown to impair antitumor responses of NK cells, suggesting that tumor environment would share a mechanism of immunosuppression similar to that occurring in fetal–maternal tolerance [43]. Another mechanism that has been implicated in the suppression of NK cells in tumor environment involves production of adenosine. Under physiological conditions, adenosine is produced by ectonucleotidases expressed on T-reg lymphocytes to modulate the immune response, particularly in inflammatory processes [44]. However, expression of CD39 and CD73 ectonucleotidases has also been reported on EOC cells [45,46]. Thus, excessive production of adenosine, both by lymphocytes and EOC cells, could generate an immunosuppressive environment. Finally, activation of checkpoint inhibitory receptors expressed by cytotoxic lymphocytes could also halt antitumor responses. Thus, the expression of PD-L1 (programmed death-ligand 1) molecule has been reported on EOC cells and its interaction with the PD-1 (programmed death-1) checkpoint inhibitory receptor could impair NK antitumor response [47,48,49]. Although there are studies on the phenotypic modulation of NK cells and other lymphocytes in women with advanced EOC, they are still limited. Additionally, this issue requires to be frequently revisited due to the extension of knowledge on molecules and signaling pathways involved on the immune modulation of the tumor environment. Herein, we carried out flow cytometry-based analyses to reassess phenotypic modulation of the NK and T lymphocyte subsets in women with advanced EOC, moreover, to evaluate functional responses and modulation of signaling pathways involving IL-2 and IL-15 cytokines.

## 2. Material and Methods

### 2.1. Patients and Samples

For this study, we included 14 women assisted in the Women’s Hospital of the University of Campinas (Campinas, Brazil) for initial diagnosis of adnexal masses. Inclusion criteria comprised the presence of ascites and confirmation of serous histotypes (grades III–IV) or carcinomas not otherwise specified of the ovaries. Specific patient characteristics at the time of sample collection are provided in Table 1. Among the 14 ascites samples, eight ascites were positive for EOC cells as determined through cytological evaluation. Signed informed consent was obtained from all patients. The study was approved by the Research Ethics Committee of the University of Campinas (24 April 2017, #2.029.221/2017) and was registered on the Brazilian National Health Council (CAAE: 65367617.9.0000.5404). Blood samples were collected using 10 mL vacuum blood-sampling tubes containing sodium heparin (Vacuette, Campinas, Brazil). Ascites samples from patients with ovarian cancer were collected by ultrasonography-guided paracentesis, quickly transferred to 50 mL conical tubes, and added with sodium heparin (1 µL/mL of ascites; Liquemine 5000 UI/mL, Roche, Rio de Janeiro, Brazil) under sterile conditions. Initially, 1 mL of every sample of blood and 5 mL of every sample of ascites were transferred to new conical tubes and centrifuged at 600× *g* for 5 min to obtain cell-free plasma and ascites fluid, respectively. Then, the resulting supernatants were kept frozen (−30 °C) until used for soluble molecules and cytokine quantification. Subsequently, peripheral blood mononuclear cells (PBMC) were isolated by gradient centrifugation, using Ficoll-Paque Plus (GE Healthcare, Uppsala, Sweden), followed by a washing procedure performed twice (centrifuged at 600× *g* for 5 min) using a balanced salt solution (PBS; Nutricell Nutrientes Celulares Ltda, Campinas, Brazil). The cellular fraction of the ascites was isolated by centrifugation (centrifuged at 600× *g*/5 min) and red blood cells eliminated washing cell pellet with lyses solution. Cell numbers were assessed in a Neubauer chamber using acetic acid solution (2% *v*/*v* in PBS) and the trypan blue (1% *w*/*v* in PBS) exclusion method to assess viability. Replicates of the resulting cell pellets were cryopreserved in fetal bovine serum (FBS; LCG Biotecnologia Ltda, Cotia, SP, Brazil) containing 10% dimethyl sulfoxide (DMSO; Sigma, St. Louis, MO, USA), for subsequent phenotyping of the lymphocytes and evaluation of NK cell function.

### 2.2. K562 Cell Line Target Cells

The K562 (human erythromyeloblastoid) cell line, originally obtained from the American Type Culture Collection (ATCC, Rockville, MD, USA), is routinely maintained in our laboratory and periodically assessed for its usual surface markers, particularly CD45^pos^ HLA-class I^neg^ phenotype. The K562 cells are cultured in vitro in RPMI-1640 medium supplemented with 10% FBS, 2 mM L-glutamine, and replenished with fresh medium every 2–3 days.

### 2.3. Monoclonal Antibodies, Panels, General Staining Procedure and Acquisition

Flow cytometric-based assays were used according to standardized procedures [19] for the evaluation of phenotypic modulation of the lymphocyte subsets in blood and ascites of women with advanced EOC. For this end, three panels containing mixtures of fluorochrome-conjugated monoclonal antibodies (MAb, Becton Dickinson, San Jose, CA, USA) were established as follows: *Panel 1:* anti-CD56 PE-Cy7 (clone B159), anti-CD45 PE-Cy5 (clone HI30), anti-CD155 PE (clone 2H7), anti-CD39 BB515 (clone TU66), anti-CD3 APC-H7 (clone SK7), anti-CD274 APC (clone MIH4), anti-CD73 BV510 (clone AD2) and anti-PD-1 BV421 (clone MIH4). *Panel 2:* anti-CD56 PE-Cy7 (clone B159), anti-CD4 PerCP (clone Leu3A), anti-CD215 PE (clone JM7A4), anti-CD25 BB515 (clone 2A3), anti-CD3 APC-H7 (clone SK7), anti-CD127 Alexa647 (clone HIL7R-M21), anti-CD 107a BV510 (clone H4A3) and anti-CD122 BV421 (clone MIK-B3). *Panel 3:* anti-CD45 PE-Cy7 (clone HI30), anti CD8 FITC (clone OKT8), anti-CD3 APC-H7 (clone SK7), anti-CD56 APC (clone B159), anti-STAT5 BV421 (clone pY694) and anti-pS6 PE (clone PS235/PS236). Thus, the MAb panels were optimized to characterize lymphocyte profile in blood and ascites of the patients, simultaneously providing information on phenotypic modulation and functional performance of effector cells. The staining procedures involving panels 1 and 2 were performed by the incubation (30 min/8 °C, protected from light) of 0.3 × 10^6^ cells suspended in 50 µL of the MAb mixture diluted at 1:50 (*v*/*v*) in staining solution (PBS containing 2% FBS *v*/*v* and ethylenediaminetetraacetic acid, EDTA, 2 mM—Invitrogen Life Technologies, Carlsbad, CA, USA). After the incubation, cells were washed twice with PBS and the final pellets suspended for acquisition in a FACSVerse cytometer using the FACSuite software (Becton Dickinson, San Jose, CA, USA). FlowJo software (Version 10, Becton Dickinson, San Jose, CA, USA) was used for the data analysis. The lymphocyte population was identified by the FSC (*forward-scatter*) and SSC (*side-scatter*) parameters, and then FSC-Area vs. FSC-Height was used to eliminate doublets. Between 20,000 and 50,000 cells were acquired.

### 2.4. NK Cell Degranulation Assay and IL-2/IL-15 Receptors Phenotyping

The functional characteristics of NK cells were evaluated by a standardized degranulation assay based on flow cytometry [50], which allows visualization of cell surface-expressed CD107a to quantify activated NK effector cells. The method was carried out with PBMCs and ascites cells of the patients as previously described in da Silva et al. [19]. Briefly, resting and IL-2-stimulated (1000 U/1 × 10^6^ cells/mL, incubated 37 °C/5% CO_2_/18 h) PBMCs or ascites cells were co-incubated (37 °C/5% CO_2_/2 h) with target cells (K562 cell line) in a 1:1 ratio, resulting in a final density of 0.2 × 10^6^/200 µL, in U-bottom microtubes (0.6 µL, Jetbiofil, Guangzhou, China), in duplicate. Cells were spun down quickly (30× *g* for 3 min) and incubated for 2 h at 37 °C. Tubes containing effector cells without target cells were also prepared for quantification of basal expression of CD107a. After incubation, microtubes were centrifuged (600× *g* for 5 min), supernatants discarded and cell pellets suspended in 50 µL of staining solution containing MAb consistent with panel 2. Staining procedures were carried out as described above. 

### 2.5. PSTAT5 and pS6 Phosphorylation Assay

The phosphorylation status of intracellular proteins STAT5 and pS6 was evaluated in NK and CD8 lymphocytes from ascites and blood of the patients by a method based on flow cytometry. For this end, cryovials containing ascites cells or PBMC were removed from the liquid nitrogen, thawed at room temperature and washed twice with PBS (centrifuged at 600× *g*/5 min). Cell number and viability were assessed and the cell suspension was adjusted to a final density of 1 × 10^6^ cells/mL with RPMI-1640 supplemented with 10% FBS and 2 mM L-Glutamine (Nutricell Nutrientes Celulares Ltd.a, Campinas, SP, Brazil). Cells were plated (10 × 35 mm dish Sarstedt, Inc., Newton, MA, USA) and incubated overnight (37 °C/5% CO_2_/18 h). After incubation, each of the cell suspensions obtained by this procedure were split into four replicates, containing 0.5 × 10^6^ cells in V-bottom microtubes (2.0 mL, Axygen, New York, NY, EUA) to proceed with appropriate stimuli simultaneously with the staining of cell surface markers. Thus, three of the four replicates were respectively stimulated with PMA (50 ng/mL; *Phorbol 12-myristate 13-acetate*, Sigma-Aldrich, Inc. St. Louis, MI USA), IL-2 (100 ng/mL; Becton Dickinson, San Jose, CA, USA) or IL-15 (100 ng/mL; Peprotech, Rocky Hill, NJ, USA), while one of the replicates remained without stimulation, as experimental control. Subsequently, all replicates were added with the staining solution containing MAb for the labelling of cell surface molecules (Panel 3: anti-CD45 PE-Cy7, anti CD8 FITC, anti-CD3 APC-H7 and anti-CD56 APC) and incubated at 37 °C in 5% CO_2_. At the end of the appropriate incubation period for each of the stimuli (PMA/15 min, IL-2/30 min and IL-15/45 min) the cell suspensions were centrifuged (400× *g*/7 min), washed once with staining solution (centrifuged at 400× *g*/7 min) and the final pellet resuspended with 500 µL of preheated fixation buffer (Cytofix at 37 °C; Becton Dickinson, San Jose, CA, USA) and incubated at 37 °C/10 min. After fixation, cells were washed with staining solution (centrifuged at 400× *g*/7 min), supernatants were discharged and pellets were kept to proceed with the steps of permeabilization and labelling of intracellular molecules. Thus, permeabilization was carried out by gently vortex cell pellets in remained volume for, subsequently, to suspend cells thoroughly with 500 µL of −20 °C pre-cooled permeabilizing solution (Perm-buffer III, Becton Dickinson, San Jose, CA, USA), which was followed by 30 min incubation at 4 °C. After permeabilization, cells were gently washed twice with 500 µL of 8 °C pre-cooled staining solution in a centrifuge with disabled break function (centrifugation at 400× *g*/7 min/no-break). Cell pellets were finally suspended and incubated (30 min/8 °C, protected from light) in 50 µL of staining solution containing anti-STAT5 and anti-pS6 MAb (panel 3). After staining of the intracellular molecules, cells were gently washed twice with 500 µL of 8 °C pre-cooled staining solution by centrifugation with disabled break function (centrifugation at 400× *g*/7 min/no-break). Final cell pellets were suspended in 400 µL staining solution for proceeding acquisition in a FACSVerse cytometer.

### 2.6. Cytokines Assay

The presence of cytokines in blood plasma and ascites supernatant was determined by the CBA assay (Cytometric Bead Array, BD Biosciences, San Jose, CA, USA). The kit used for the analysis of cytokines was the Th1/Th2 CBA kit, specific for human IL-2, IL-4, IL-6, IL-10, tumor necrosis factor (TNF)-α, and IFN-γ. Experiment was conducted according to the manufacturer’s protocol. 

### 2.7. TGF-β, VEGF and CA125 Assays

The presence of tumor growth factor-β (TGF-β) and vascular endothelial growth factor (VEGF) in blood plasma and ascites supernatant were determined by the ProQuantum immunoassay (Invitrogen—Thermo Fisher Scientific, Rockford, IL, USA), which is based on analyte specificity of antibody–antigen binding with the signal detection and amplification capabilities of qPCR. The presence of CA125 molecule in blood plasma and ascites supernatant was determined in an automated chemiluminescence analyzer using the Liaison CA125 II kit (DiaSorin S.p.A., Saluggia, VC, Italy). All the assays were conducted according to the manufacturer’s protocol. 

### 2.8. Statistical Analysis

Comparison of variables within groups was performed using the Student’s *t*-test for independent samples. Multi-comparison analysis of variables was performed by ANOVA followed by a post hoc multiple comparison test. The level of significance was set at *p*-value < 0.05. Statistical analysis was carried out using Prism software version 9 (GraphPad Software, San Diego, CA, USA).

## 3. Results 

### 3.1. Phenotypic Modulation of NK and T Lymphocytes 

**Figure 1:**
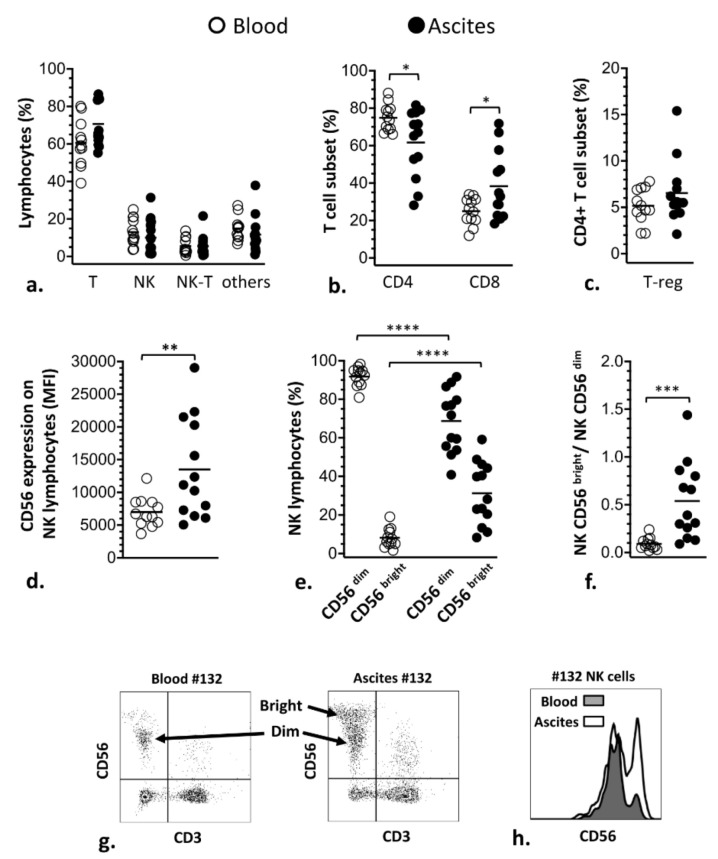
Comparison of the contents of lymphocyte subsets in blood (white circles) and ascites (black circles) from women with advanced EOC. (**a**) Frequencies of T, NK, NK-T lymphocytes; “others” include lymphocytes that were negative for the markers used in cytometry, which mostly comprises B-lymphocytes. (**b**) Frequencies of CD4 and CD8 T-subsets. (**c**) Frequencies of T-reg subset. (**d**) Mean fluorescence intensity (MFI) of the CD56 molecule on NK lymphocytes. (**e**) Frequencies of CD56^dim^ and CD56^bright^ subsets of NK lymphocytes. (**f**) Ratio of CD56^bright^/CD56^dim^. (**g**) Representative dot plots and histogram (**h**) comparing CD56 fluorescence intensity in NK cells from blood and ascites of women with advanced EOC. Statistical analyses were performed by unpaired two-tailed Student’s *t*-test, and *p*-values (* *p* < 0.05, ** *p* < 0.01, *** *p* < 0.001 and **** *p* < 0.0001) indicate significant statistical differences.

The contents of NK and T lymphocyte subsets in ascites of women with advanced EOC were assessed and compared to lymphocytes from their blood to characterize phenotypic modulation related to the tumor environment. Initially, our results showed that, although frequencies of T (CD3^+^CD56^−^), NK (CD3^−^CD56^+^) and NK-T (CD3^+^CD56^+^) cells within total of lymphocytes (Figure 1a) were similar between ascites and blood of the patients, frequencies of T subsets (CD3^+^) varied. Thus, the T-CD4^+^ subset was significantly lower in ascites, whilst the T-CD8^+^ was higher compared with blood (Figure 1b). Additionally, frequency of T-regulatory (T-reg, CD4^+^CD25^+^CD127^−^) subset showed a tendency to be increased in ascites (Figure 1c). NK lymphocytes also underwent phenotypic modulation in ascites, where expression of CD56 molecules on NK cells became significantly higher than in blood (Figure 1d), causing, additionally, a significant increase in the frequency of C56^bright^ NK cells (Figure 1e,f).

**Figure 2:**
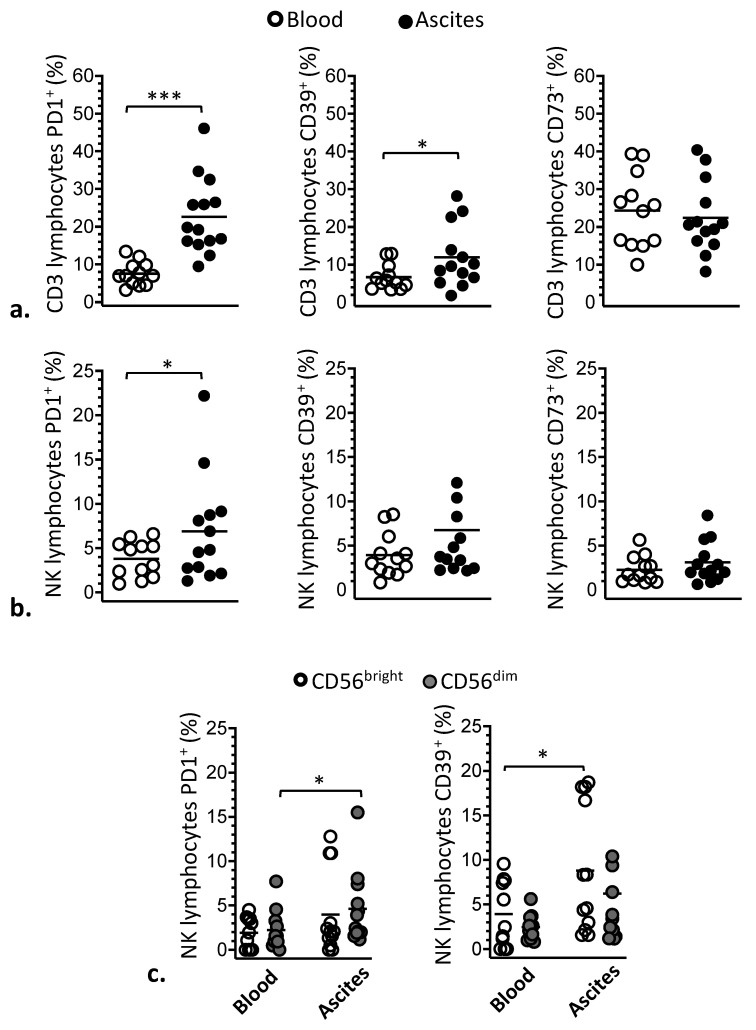
Expression of PD1, CD39 and CD73 molecules on CD3 (**a**) and NK (**b**) lymphocytes from blood (white circle) and ascites (black circle) of women with advanced EOC. Expression of PD1 and CD39 molecules were also assessed in CD56^bright^ ((**c**). white circles) and CD56^dim^ ((**c**.) grey circles) subsets of NK lymphocytes from blood and ascites of the patients. Values are presented as percentage of positive cells and mean. Statistical analyses were performed by unpaired one-tailed Student’s *t*-test; * *p* < 0.05 and *** *p* = 0.0001 values indicate significant statistical differences.

**Figure 3:**
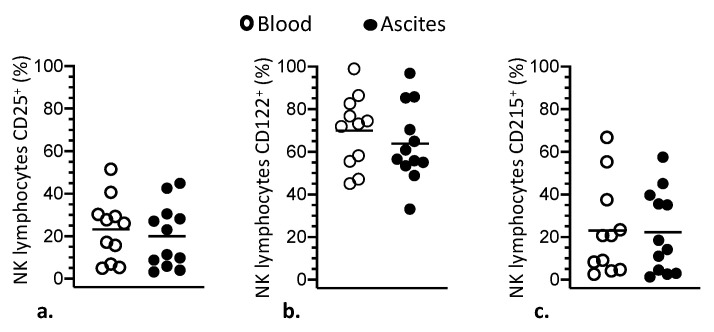
Comparison of blood (white circle) and ascites (black circle) of women with advanced EOC in relation to the expression of CD25 (**a**), CD122 (**b**) and CD215 (**c**) molecules on NK lymphocytes, which comprise IL-2 and IL-15 receptors. Values are presented as percentage of positive cells and mean.

Due to the importance of these molecules on the functional modulation of immune response in tumor environment, ascites and blood of patients were also compared in relation to the expression of the immune checkpoint inhibitory receptor PD-1 and the ectonucleotidases CD39 and CD73 on lymphocytes (Figure 2). As a result, frequencies of NK and T lymphocytes expressing PD-1 were significantly higher in ascites compared with blood (Figure 2a,b). Similarly, it was found that ascites had significantly higher percentages of T lymphocytes expressing CD39 molecules than blood of the patients (Figure 2a). Facing these results, the distribution of PD1 and CD39 molecules was also assessed in CD56^bright^ and CD56^dim^ NK cell subsets. Thus, the frequencies of CD56^dim^ NK cells expressing PD1, as well as CD56^bright^ expressing CD39, were significantly higher in ascites than in blood of the patients (Figure 2c). Ascites and blood of women with advanced EOC were further compared for the expression of IL-2 and IL-15 cytokine receptors, CD25, CD122 and CD215 molecules, on NK cells, which were found to be similar in these two compartments (Figure 3). Likewise, the distribution of CD25, CD122 and CD215 molecules on CD56^bright^ and CD56^dim^ NK cells subsets followed the same pattern found for the whole NK cell population (Supplementary Figure S1).

### 3.2. Degranulation of CD56^bright^ and CD56^dim^ NK Lymphocyte Subsets

**Figure 4:**
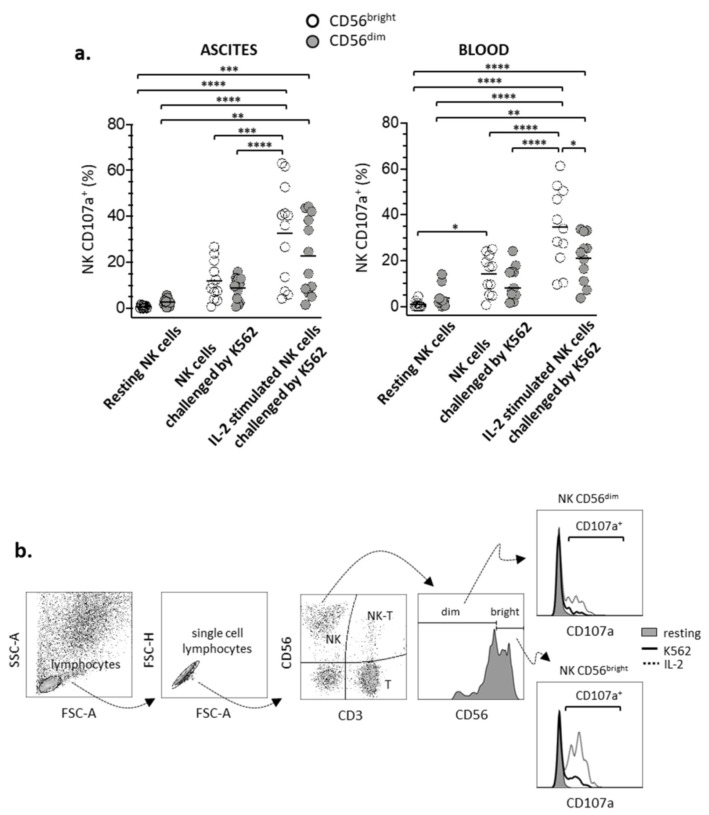
(**a**) Comparison of degranulation of CD56^bright^ (white circles) and CD56^dim^ (gray circles) NK lymphocytes from ascites and blood of women with advanced EOC. Degranulation was evaluated by the expression of the CD107a molecule on NK cells under resting, and after IL-2 stimulation overnight, while co-incubated (2 h, ratio 1:1) with K562 target cells. Overnight (18 h) stimulation with rhIL-2 (1000 UI/mL) was conducted in RPMI-1640 medium supplemented with FBS (10%) and L-glutamine (2 mM). Values are presented as a percentage of positive cells and mean. Resting NK cells indicates the “background” expression of CD107a on NK lymphocytes in the absence of target cells K562. (**b**) Flow cytometry-based analysis of NK cell degranulation. To determine CD107a expression, NK cells were gated from the whole lymphocyte population, based on their expression of CD56 molecule and absence of CD3; subsequently, CD56 bright and dim subsets were gated from the whole NK population. Statistical analyses were performed by ANOVA followed by Tukey’s multiple comparisons test and *p*-values (* *p* < 0.05, ** *p* < 0.01, *** *p* < 0.001, **** *p* < 0.0001 on the brackets) indicate significant statistical differences.

The functionality of CD56^bright^ and CD56^dim^ NK subsets from ascites and blood of women with advanced EOC was evaluated by the ability of these lymphocytes to undergo degranulation when challenged in vitro by standardized target-cells, as well as after in vitro stimulation with IL-2. Degranulation action in NK lymphocytes elicits expression of CD107a molecule on cell surface, which can be quantified by flow cytometry. Figure 4 (and Supplementary Table S1) shows the percentages of CD56^bright^ and CD56^dim^ NK cells expressing CD107a molecule and compares these two subsets in relation to their degranulation capacity, as well as responsiveness to stimulation with IL-2. The results show that the CD56^bright^ NK subset, either from ascites or blood, when stimulated with IL-2, increased significantly the percentages of cells expressing CD107a molecule than non-stimulated cells that were just challenged by target cells. In contrast, IL-2 treatment was not effective in increasing significantly the percentages of NK-expressing CD107a in CD56^dim^ subset. These results indicate that CD56^brigth^ NK were more responsive to stimulation with IL-2 than CD56^dim^ NK lymphocytes.

### 3.3. STAT5 and pS6 Phosphorylation in Lymphocyte

**Figure 5:**
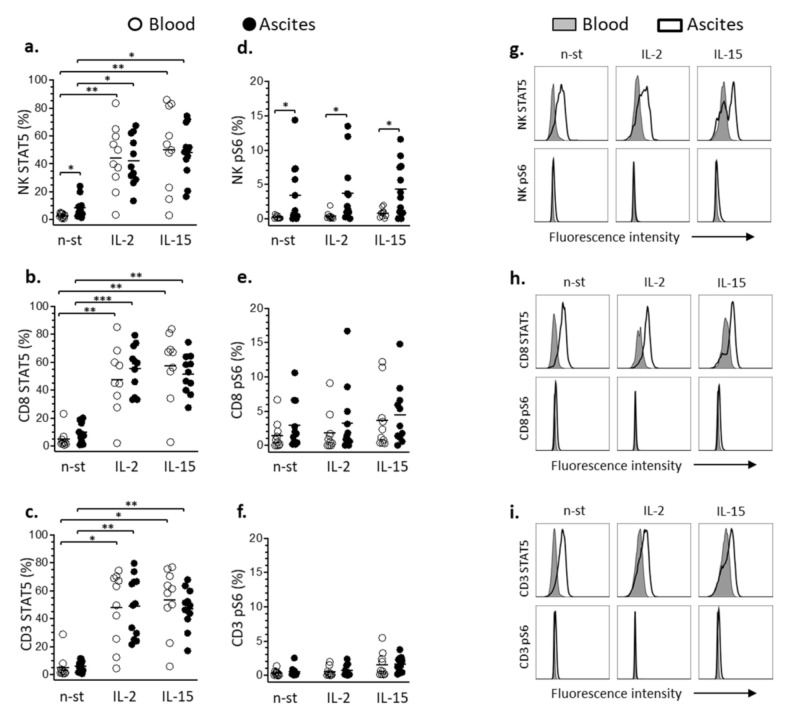
Comparison of phosphorylation of proteins STAT5 and pS6 in NK (**a**,**d**), CD8 (**b**,**e**) and CD3 (**c**,**f**) lymphocytes subsets from blood (white circles) and ascites (black circles) of women with advanced EOC. Protein phosphorylation was evaluated in non-stimulated (n-st) and after stimulation of blood and ascites cells with IL-2 (100 ng/mL, 30 min) or IL-15 (100 ng/mL, 45 min). Stimulation was conducted in RPMI-1640 medium supplemented with FBS (10%) and L-glutamine (2 mM). Values are presented as percentage of positive cells and mean. Statistical analyses were performed by ANOVA followed by uncorrected Fisher’s LSD multiple comparisons test and *p*-values (* *p* < 0.05, ** *p* < 0.01, *** *p* < 0.001 on the brackets) indicate significant statistical differences. Representative histograms comparing fluorescence intensities of STAT5 or pS6 phosphorylated proteins in NK (**g**), CD8 (**h**) and CD3 (**i**) lymphocytes from blood (gray) and ascites (white) of women with advanced EOC.

The phosphorylation state of STAT5 and pS6 proteins of NK, T-CD3^+^ and T-CD8^+^ lymphocytes from ascites of women with advanced EOC was assessed and compared with lymphocytes from their own blood to characterize phenotypic modulation of JAK-STAT5 and mTOR-S6 signaling pathways related to the tumor environment. Protein phosphorylation was evaluated in non-stimulated and stimulated cells from ascites and blood either with IL-2 or IL-15. Thus, it was initially observed that non-stimulated lymphocytes from ascites had significantly higher percentages of NK cells with phosphorylated STAT5 protein than lymphocytes from blood of the patients. Additionally, treatment of the cells with IL-2 or IL-15 increased significantly phosphorylation of STAT5 in NK, T-CD3^+^ and T-CD8^+^ lymphocytes from ascites and blood (Figure 5a–c). In contrast, although the percentages of NK lymphocytes with phosphorylated pS6 were significantly higher in ascites than in blood, treatment of the cells with IL-2 or IL-15 did not cause additional phosphorylation of pS6 in any of the evaluated lymphocyte populations (Figure 5d–f). This observation was confirmed by the fluorescence intensities of phosphorylated pS6, since it remained unchanged between cytokine-stimulated and non-stimulated lymphocytes (Figure 5g–i).

### 3.4. Soluble Signaling Molecules Profile in Ascites and Blood

**Figure 6:**
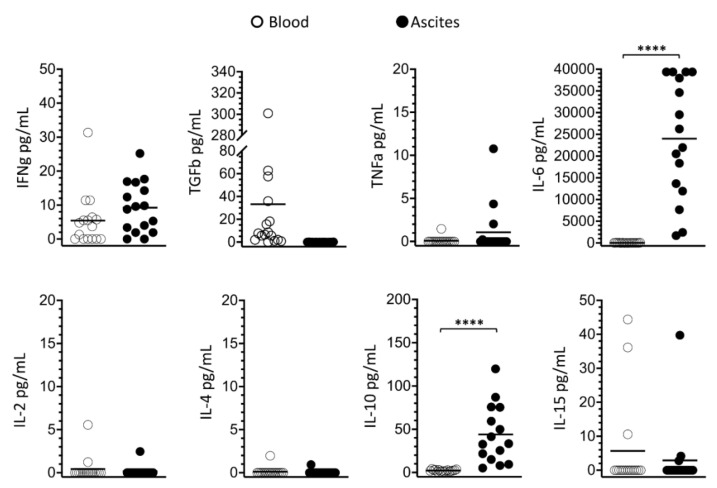
Comparison of soluble signaling molecules (IL-2, Il-4, IL-6, IL-10, IL15, IFNg, TNFa, TGFb) in blood (white circles) and ascites (black circles) of women with advanced EOC. Values are presented as percentages and mean. Statistical analyses were performed by unpaired two-tailed Student’s *t*-test, and *p*-values (**** *p* < 0.0001) indicate significant statistical differences.

The concentrations of soluble signaling molecules IL-2, IL-4, IL-6, IL-10, IL-15, IFN-γ, tumor necrosis factor (TNF)-α, tumor growth factor (TGF)-β, cancer antigen 125 (CA125) and VEGF were determined in ascites supernatants and plasma of women with advanced EOC. Ascites was found to have significantly high amounts of IL-6 and IL-10, while just traces of these molecules were detected in the plasma (Figure 6). As expected for patients with advanced EOC, CA125 was elevated in the plasma (2099 ± 1820 U/mL) and its concentration in ascites (29,876 ± 29,167 U/mL) was significantly higher (*p* < 0.0001), overcoming plasma in more than ten times. No significant differences were observed between VEGF concentrations in blood (1161.9 ± 2108.0 pg/mL) and ascites (884.2 ± 1222.6 pg/mL).

## 4. Discussion

In this study, we carried out flow cytometry-based analysis to assess NK and T lymphocyte subsets in ascites and blood of women with advanced EOC to characterize lymphocyte phenotypic modulation related to the tumor environment. In this context, we were particularly successful in assessing the phosphorylation responses of STAT5 and pS6 proteins in NK and T lymphocytes, as well as the functional performance of the CD56^bright^ and CD56^dim^ NK subsets. Additionally, we confirmed EOC-associated ascites as an inflammatory environment, further demonstrating altered expression of the PD-1 and CD39 molecules within NK and T lymphocytes.

In contrast to previous reports showing that NK cells are dysfunctional in many human solid malignancies, including ovarian cancer [51,52,53,54], we recently showed that NK cells from EOC-associated ascites could display either improved or impaired functionality. Accordingly, we reported that NK cells from ascites positive for EOC cells, i.e., presence of carcinomatosis as confirmed by cytologic results, displayed poor degranulation responses and were hyporesponsive to IL-2 stimulation, while NK cells from ascites negative for the presence of EOC cells displayed robust degranulation and responsiveness to IL-2 [19]. Herein, the results not only confirmed our previous findings, but also revealed important functional differences between the CD56^bright^ and CD56^dim^ subsets of NK cells in EOC-associated ascites. Therefore, our present study points out that CD56^dim^ NK cells seem to be more susceptible to the suppressive mechanisms operating in tumor environment, while CD56^bright^ cells are more responsive to the stimulatory cytokines and, consequently, their functional performance can be improved.

As aforementioned, the CD56^bright^ subset of conventional NK cells has conceptually been categorized as lymphocytes that play a role in modulation of the immune response, given their cytokine-producing characteristics, array of expressed receptors and low cytotoxic capacity [22,55]. Nevertheless, a number of studies have shown that highly cytotoxic NK cells can be generated by long-term in vitro culture of isolated NK cells or PBMCs maintained under continuous treatment with stimulatory cytokines such as IL-2 or IL-15 [56,57,58,59,60]. Interestingly, the cellular products obtained as a result of such a culture processes are, frequently, enriched CD56^bright^ NK cell suspensions. Moreover, recent studies have shown that antitumor cytotoxic response of CD56^bright^ NK cells can be enhanced by the IL-15 cytokine, not only in vitro but also in vivo. Thus, CD56^bright^ NK cells from multiple myeloma patients, after being primed through their IL-15 receptor, displayed antitumor responses against autologous blasts in vitro, and controlled leukemia development in vivo in a murine xenograft model [33]. Additionally, infusion of IL-15 in patients with cancer expanded CD56^bright^ subset of NK lymphocytes and also elicited their antitumor cytotoxicity [34].

Nevertheless, the biological implication of the presence of CD56^bright^ NK cells in EOC-associated ascites was previously explained in terms of an immunomodulatory effect similar to that occurring in fetal maternal tolerance [61,62]. In this context, Belisle et al. [61,63] showed that, similar to the decidua in pregnancy, in EOC-associated ascites occur preferential binding of CA125 to the CD16 surface molecules of CD56^dim^ NK cells, as well as the accumulation of CD56^bright^ NK cells. Additionally, the CD56^bright^ phenotype is frequently associated with VEGF-producers immature NK cells, which is consistent with the development of a tumor-supportive environment [62,64,65]. Likewise, our results confirmed the occurrence of large amounts of CD56^bright^ NK cells in EOC-associated ascites. However, based on the current knowledge that antitumor responses can be elicited in the CD56^bright^ subset [33,34], and in the results herein reported, we wonder whether accumulation of CD56^bright^ NK cells in ascites could be used to improve therapies designed to overcome immunosuppression in the ovarian cancer tumor environment.

Based on our previous findings [19], we hypothesized that the hyporesponsiveness to IL-2, which we observed in NK cells of certain ascites from women with epithelial ovarian cancer, could be a consequence of impairment of the IL-2 and IL-15 signaling pathways. Thus, our goals in the present study included to compare NK cells from blood and ascites of women with EOC in relation to the expression of IL-2 and IL-15 receptors and JAK-STAT5 and mTOR-S6 pathways activation. Favorable to the use of NK cells as therapeutic target in ovarian cancer treatment, our results showed that ascites, as a tumor environment, did not affect the expression of the CD25, CD122 and CD215 molecules, which comprise the receptors for IL-2 and IL15 cytokines. Furthermore, our results showed not only activation of the JAK-STAT5 signaling pathway in NK cells from EOC-associated ascites, but also that these lymphocytes remained responsive to cytokine treatment. Interestingly, activation of the JAK-STAT5 signaling pathway in NK cells might have been mediated upon direct cell contact, considering that IL-2 and IL15 cytokines were not detected in ascites, and in spite of the large amounts of IL-10 in this tumor environment. Gotthard et al. [39] showed that IL-10 and other cytokines frequently present in tumor environment affect the JAK-STAT5 pathway by decreasing the phosphorylation state of STAT5 protein, while enhancing expression of VEGF of NK cells. Under physiological conditions, this mechanism ensures tissue-specific properties for uterine NK cells; however, in the tumor environment, such a mechanism would contribute to angiogenesis and tumor development [39]. Similar to the STAT5 protein in the JAK-STAT signaling pathway, the phosphorylation state of the pS6 protein has also being used as activation marker of the mTOR signaling pathway [38,40]. Thus, our findings revealed that in vitro treatments of leukocytes from blood or ascites of women with EOC with IL-2 or IL-15 did not cause additional phosphorylation in pS6 of NK or T-CD8 lymphocytes. However, the proportion of NK cells displaying activation of the mTOR signaling pathway were significantly higher in ascites than in blood of women with EOC. Interestingly, we found low pS6 phosphorylation in NK cells from blood, which matched the presence of significantly higher concentration of TGF-β in blood of the patients compared to ascites, and the well-known inhibitory effect of TGF-β on the mTOR signaling pathway [40,54,66,67].

Finally, our results confirm EOC-associated ascites as an inflammatory tumor environment characterized by the presence of IL-6 [19,52,68]. However, this inflammatory profile contrasts with the presence of immunosuppressive elements, such as large amounts of the suppressor cytokine IL-10. Additionally, phenotypic modulation of lymphocytes in ascites indicates development of self-regulating mechanisms, aimed to control an exacerbated immune response under chronic stimulatory conditions. Accordingly, our results showed that, in contrast to blood of the patients, ascites accumulated NK and T lymphocytes expressing PD-1 molecule, which is a hallmark for development of lymphocyte exhaustion processes [69,70]. In addition, the proportion of T lymphocytes expressing CD39 molecule was also significantly larger in ascites than in blood of the patients, which is implicated in the local development of self-regulating mechanisms based on adenosine production [44,45].

## 5. Conclusions

EOC-associated ascites allows studies to investigate lymphocyte phenotype modulation in the tumor environment, fostering important insights into the mechanisms involved in immune responses against ovarian cancer, tumor evasion and immunosuppression. In this context, the inflammatory profile found in EOC-associated ascites contrasts with the presence of immunosuppressive elements and development of cellular self-regulating mechanisms.

## Figures and Tables

**Table 1:** Characteristics of epithelial ovarian cancer (EOC) patients included in the study at the time of ascites sampling. Staging classification followed FIGO Committee on Gynecologic Oncology guidelines.

Patient’s Code	Age (Years)	FIGO (Stage)	Histotype	Ascites Cytology for EOC Cells
101	59	III	HGS	Negative
104	53	III	HGS	Negative
106	65	IV	HGS	Positive
107	70	III	HGS	Positive
108	51	III	HGS	Negative
112	71	III	HGS	Positive
113	33	III	LGW	Negative
115	78	III	HGS	Negative
116	70	III	NOS	Positive
121	82	III	NOS	Positive
122	52	III	HGS	Positive
127	61	III	HGS	Negative
130	62	III	HGS	Positive
132	40	III	HGS	Positive

HGS = High-grade serous; LGS = Low-grade serous; NOS = not otherwise specified.

## Supplementary Materials

The following are available online at https://www.mdpi.com/article/10.3390/cells10071702/s1, Figure S1: Expression of CD25, CD122 and CD215 molecules on CD56bright and CD56dim NK lymphocytes, Table S1: Degranulation of CD56bright and CD56dim NK lymphocytes.
