# Supplementary material for: Ovarian Cancer-Associated Ascites Have High Proportions of Cytokine-Responsive CD56bright NK Cells
Source: Cells. 2021 Jul 6;10(7):1702. doi: 10.3390/cells10071702 (PMC8306021; doi:10.3390/cells10071702)
Supplement: Supplementary file 1 [file cells-10-01702-s001.zip › cells-1215653-supplementary.pdf]

Supplementary table 1. Comparison of degranulation of CD56^{bright} and CD56^{dim} NK lymphocytes from blood and ascites of women with advanced EOC. Degranulation was evaluated by the expression of the CD107a molecule on NK cells under resting, and after IL-2 stimulation overnight, while coincubated (2 h, ratio 1:1) with K562 target cells. Overnight (18h) stimulation with rhIL-2 (1000 UI/mL) was conducted in RPMI-1640 medium supplemented with FBS (10%) and L-glutamine (2 mM). Values are presented as mean percentage of CD107a positive NK cells and ±standard deviation. Resting NK cells indicates the "background" expression of CD107a on NK lymphocytes in the absence of target cells K562. Statistical analyses were performed by ANOVA followed by Tukey's multiple comparisons test, and numbers labeled with equal letters did not differed significantly, while different letters indicate significant statistical differences for $p < 0.05$.

Blood						Ascites					
Resting NK cells		NK + K562		NK + IL2 + K562		Resting NK cells		NK + K562		NK + IL2 + K562	
CD56^{br}	CD56^{dim}	CD56^{br}	CD56^{dim}	CD56^{br}	CD56^{dim}	CD56^{br}	CD56^{dim}	CD56^{br}	CD56^{dim}	CD56^{br}	CD56^{dim}
0.9[a]	3.8[a]	14.1[ac]	10.6[ac]	34.6[d]	21.2[bc]	0.7[a]	2.6[a]	11.8[ac]	8.7[ac]	32.8[bd]	22.8[bc]
±1.5	±4.5	±8.5	±7.3	±17.1	±10.5	±0.7	±1.5	±8.4	±5.2	±21.2	±16.8

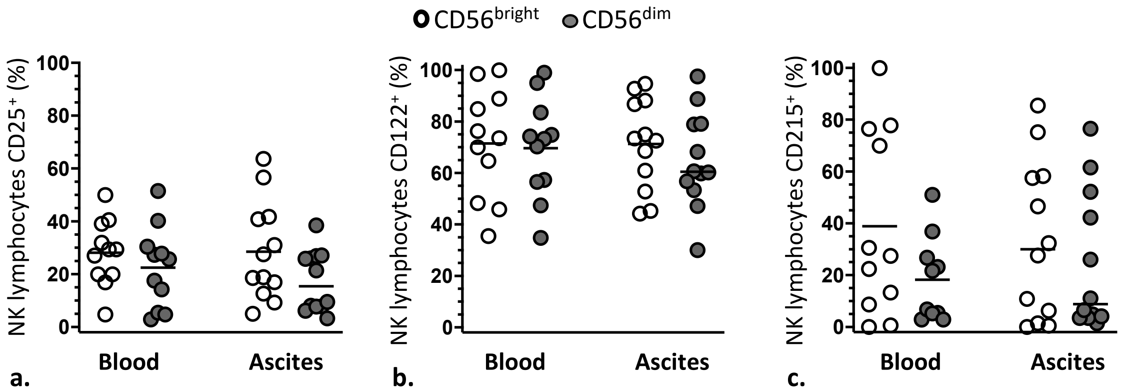

Supplementary figure 1. Comparison of blood and ascites of women with advanced EOC in relation to the expression of CD25 (a), CD122 (b) and CD215 (c) molecules on CD56bright (white circle) and CD56dim (grey circle) NK cell subsets, which comprise IL-2 and Il-15 receptors. Values are presented as percentage of positive cells and mean.
